# ‘Disabled joy is resistance’: Insights and recommendations from social psychology on reducing ableism

**DOI:** 10.1111/bjso.12893

**Published:** 2025-04-28

**Authors:** Siân E. Jones

**Affiliations:** ^1^ Division of Psychology, Sociology and Education Queen Margaret University Edinburgh UK

**Keywords:** ableism, anti‐ableism, disability, social identity, stereotyping, structural inequality

## Abstract

Ableism, encompassing discrimination and social oppression of disabled people, which results in their marginalization, persists as a significant global barrier to equity and inclusion. This paper explores how social psychological research can inform strategies to combat ableism by analysing the social processes that underlie ableist attitudes and actions. Social Identity Theory speaks to the role of identity in countering the marginalization of disabled people, while the Stereotype Content Model helps us to understand the origins of the stigma often attached to ‘disability’. Research in these two fields is reviewed alongside discursive research in social psychology, to determine how to work towards a more anti‐ableist society. Based on my experience as a social psychologist and disabled person, and by integrating theoretical insights with practical applications, this paper advocates for a multi‐level social psychological approach to building anti‐ableist spaces, emphasizing educational and social policy recommendations

Disability is pain, struggle, brilliance, abundance, and joy. (Wong, 2020, p. xxii)

Life sometimes seems focused on health needs. Yet I live with joy. (Hancock, 2020, p. 317)

Disability is not often seen in the same sentence as joy. More quickly, one can find a*bleism* cited alongside disability. Ableism is ‘stereotyping, prejudice, discrimination, and social oppression toward’ disabled people (Bogart & Dunn, 2019, p. 652). Examples of this may be found in the negligible representation of disabled people in mainstream media vis‐a‐vis non‐disabled people (e.g. Hayden & Prince, 2023) or the global increase in the number of ableist hate crimes (e.g. Organization for Security & Cooperation in Europe (OSCE), 2022). This paper considers how social psychological research may inform (a) work towards equity for disabled people and (b) ways to see disabled joy as resistance against unhelpful stereotypes. From my position as an apparently^1^ and non‐apparently disabled academic, I review social psychological research from several perspectives to reconceptualize mainstream perceptions of disabled people and to draw policy recommendations.

## (RE‐)FRAMING DISABILITY

According to the Equality Act Section 6 (2010), you are disabled if you ‘have a physical or mental health condition or illness that has lasted or is expected to last 12 months or more’ and ‘the condition and/or illness reduces the ability to carry out day‐to‐day activities’. In the United Kingdom, in 2021/22, 16 million people were estimated to be disabled (Department for Work & Pensions (DfWP), 2024), and the employment rates were 53.0% for disabled people and 81.6% for non‐disabled people (DfWP, 2024). In light of continued discrimination towards disabled people, the United Nations Conventions on the Rights of Disabled People [UNCRPD] (United Nations, 2006) affirms the human rights of disabled people, among other rights, to ‘enjoy their inherent right to life on an equal basis with others’ (Article 10), to have ‘equal access to primary and secondary education, vocational training, adult education and lifelong learning’ (Article 24) and to have ‘equal access to the workplace’ (Article 27). Further, it asserts that member countries should ‘combat stereotypes and prejudices’ (Article 8). This paper explores how social psychology can help us realize those rights more fully.

The Equality Act (2010) sees disability as a condition or illness that reduces one's capacity for everyday activities. Alternative perspectives have been constructed by disability activists (e.g. Oliver, 1983). The first alternative was the ‘social model’ of disability. This model challenges the medical perspective, which views impairments solely as health conditions (Shakespeare, 2013). Instead, in the social model, ‘impairment’ refers to sensory, physical or intellectual characteristics, while disability' pertains to the environmental restrictions or societal disadvantages that are consequences of those impairments.

Contemporary perspectives on disability, such as *Critical Disability Studies* (CDS), suggest that disability is more nuanced than a purely medical or social issue (Meekosha & Shuttleworth, 2009). Affirmative models advocate for a society that equally values and celebrates disabled and non‐disabled people (Swain & French, 2000). This paper rests on the assumption that it is the affirmative model of disability that is worth working towards because it forefronts the weight of social and psychological factors in reducing ableism, which we hold in our collective power to change.

## SOCIAL PSYCHOLOGY RESEARCH AND DISABLED PEOPLE

Since the 1950s, social psychology has been concerned with reducing prejudice and discrimination. Ableism is no different in this regard. At one level, social psychology may be criticized for focusing on prejudice reduction interventions that induce empathy rather than addressing structural inequalities (Nario‐Redmond, 2020). This has included attempts to simulate disability (‘experience the deficit!’), such as wearing blindfolds to simulate sensory impairments or using wheelchairs to ‘experience’ mobility impairment. Such simulations demonstrably fail to influence disability attitudes and can leave people feeling helpless, confused and vulnerable, and some participants became less keen to work on equality initiatives (Nario‐Redmond et al., 2017). Moreover, attitude‐based interventions have frequently failed to evaluate the perspectives of disabled people, sometimes collecting data from them and then discarding it (e.g. Grütter et al., 2017) or otherwise not writing about the disability identity of participants in research design or reporting (e.g., Timmons et al., 2024). At another level, social psychology *has* addressed these concerns by exploring what it is about ‘disability’ that encourages people to engage in collective action towards social change for disabled people. Here, I review research concerning stereotypes about disability and then research on disability identity and voice.

### Disability and identity: the view from outside

A large body of social psychological research on disability looks at the stereotypes made by non‐disabled people. The Stereotype Content Model (SCM) (Fiske et al., 2021) posits that stereotypes about social groups rest on two dimensions: warmth (e.g. kindness, openness) and competence (e.g. ability, skilfulness). Within this framework, disabled people are generally perceived as ‘warm but incompetent’ (e.g. Wu & Fiske, 2019). For instance, they are often viewed as unsuitable for high‐responsibility roles but as amicable colleagues (Rohmer & Louvet, 2016).

However, studies reporting the ‘warm but incompetent’ stereotype primarily focused on wheelchair users, who were often treated as representative of all disabled people (e.g., Rohmer & Louvet, 2012). To address that issue, Canton et al. (2022) looked at variations in stereotype content across different types of impairment. Pity was the most frequently associated emotion. Similarly, Pelleboer‐Gunnink et al. (2019) found that ‘in need of help, friendly’ and ‘unintelligent’ were prominent stereotypes of intellectually disabled people. These echo (unfounded) stereotypes of employers, who are often fearful of employing disabled people (see Bonaccio et al., 2020) and which may explain the employment gap cited above. Other research has shown that participants' implicit (non‐conscious) attitudes towards disabled people are also largely negative (Antonopoulos et al., 2023; Granjon et al., 2024) and that this includes the stereotypes of professionals working with disabled people (Friedman, 2023). In this vein, Rohmer and Louvet (2012) showed that at a conscious (explicit) level, participants judged disabled people as warmer but less competent than non‐disabled people. At the implicit level, disabled people were associated not only with less competence than non‐disabled people but also with less warmth. In sum, stereotyping research shows that many people hold a deficit view of disability. It highlights the pervasiveness of deficit, medical model thinking and fear and taboo. The findings reveal a need for occupational policy that recognizes a view of disability in line with the social and affirmative models. Managers need policy support to create environments in which disabled people are affirmed and where deficit‐based ideologies are actively disrupted (e.g. Whalen Smith et al., 2024).

### Disability and identity: the view from inside

Not talking about disability is a privilege that generally may only be enjoyed by non‐disabled people. In contrast to the research above, looking at the negative stereotyping of disabled people, other research in social psychology has looked at how disability activists have galvanized positive social identities.

Social Identity Theory (SIT) (Tajfel & Turner, 1979) accounts for the interplay between individual and group‐level psychological processes. In other words, similarly to the social and affirmative models of disability, SIT rejects a psychology of personhood grounded solely in individuality and underlines the criticality of group processes (Dirth & Branscombe, 2018). SIT theorizes that group members strive for a positive self‐concept through their membership. It posits that when a group is stigmatized, minoritized group members manage that stigma by either pretending to be a majority group member or by seeing their minoritized identity positively (Tajfel & Turner, 1979). Accordingly, SIT recognizes how minoritized group members construct dynamic and contextualized identities.

Developing a positive shared identity is the key to a social identity approach. However, having an impairment—and consequently holding a stigmatized identity—presents unique challenges to identity formation (Andrews et al., 2019; Bogart & Nario‐Redmond, 2019). People with an acquired impairment may distance themselves from a disability identity (Dunn & Burcaw, 2013), while people who are non‐apparently disabled may strategically choose whether to disclose their condition (e.g. Lindsay & Fuentes, 2022). Disabled *self‐identification* has been defined as the ‘extent to which one feels shame or takes disability pride; the degree to which one has integrated disability into one's sense of self; and the type of contact, camaraderie, and engagement one has with the larger disability community’ (Forber‐Pratt et al., 2019, p. 120). It is this shared social identity, rather than the reduction of disability to a medical concern, that is critical for enhancing well‐being (e.g. Bat‐Chava, 1994; Bogart, 2014; Bogart & Nario‐Redmond, 2019). An epitome of this may be seen in Cooper et al. (2021). These researchers found a positive relationship between the number of positive attributes their autistic participants associated with autism and their collective self‐esteem to the extent that they identified with other autistic people.

**FIGURE 1:**
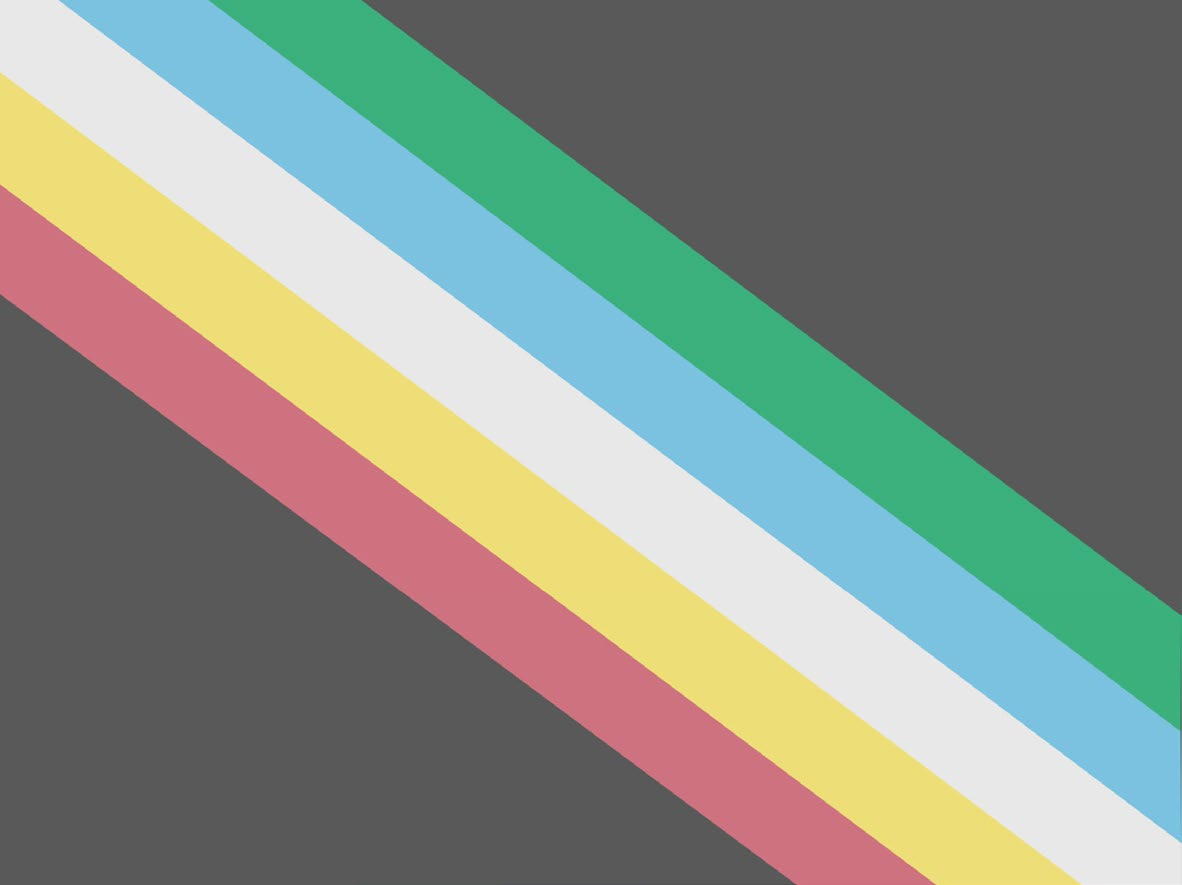
Disability pride flag. Disability pride is celebrated globally in July. Ann Magill, CC0, via Wikimedia Commons.

For disabled people, affirming a disability identity is linked to numerous benefits, such as greater life satisfaction, higher self‐esteem, increased self‐efficacy, more substantial social support, greater disability pride and lower levels of psychological distress (Bogart, 2014; Bogart et al., 2018; Davies et al., 2024; Shmulsky et al., 2021; Zapata, 2022). Factors such as experiencing ableism and being congenitally or severely disabled are predictors of identifying as disabled and embracing disability pride (Bogart, 2014; Bogart et al., 2018). Beyond this, Dirth and Branscombe (2019) showed that disabled participants' perceptions of the pervasiveness of ableist discrimination, alongside espousing social model framing of disability, predicted the perception that such discrimination was illegitimate. Moreover, Nario‐Redmond et al. (2012) showed, in line with other social identity research (e.g. Ellemers et al., 1997), that among their sample, identification as a disabled person was a strong and sole predictor of group‐level strategies to enhance equality for disabled people. Participants identifying more highly were more ready to work for social change and disability rights, reported more disability pride, and were more likely to value the experience of being disabled. As discussed later, disabled people are one of the several agents of change in reducing ableism (Figure 1).

Nonetheless, the arena which leads the way in social psychology when it comes to including the voice of disabled people is discursive psychology (DP). This approach sees disability as socially constructed and looks at how disability is talked about and ableism is re‐produced in everyday life (e.g. Lester et al., 2024). DP reclaims and redefines ableism through the voice of the disabled community as follows:

a system of assigning value to people's bodies and minds based on societally constructed ideas of normality, productivity, desirability, intelligence, excellence, and fitness. These constructed ideas are deeply rooted in eugenics, anti‐Blackness, misogyny, colonialism, imperialism, and capitalism. This systemic oppression leads to people and society determining people's value based on their culture, age, language, appearance, religion, birth or living place, ‘health//wellness’, and/or their ability to satisfactorily re/produce, ‘excel’ and ‘behave.’ You do not have to be disabled to experience ableism. (Lewis, 2022, online)

In this vein, Calder‐Dawe et al. (2019) report the ableist non‐disabled person's gaze on disability. In interviews with 35 disabled young people and their parents, the researchers revealed the erasure of participants' impairments and the everyday ableist intrusions they experienced. This included the frequent query, ‘What's wrong with you?’ which I am also commonly asked, and which is laden with ableist supposition about what bodies ‘should’ be like. For interested readers, further exploration of the discursive psychology approach to constructing our experiences of disability is given by Jammaers and Fleischmann (2024) on ableist micro‐aggressions and by O'Reilly (2021) on how parents construct paediatric mental health conditions as a burden. Other social constructionist work uses conversational analysis to examine how agency and competence are attributed (or not) to people with intellectual disabilities (Antaki & Crompton, 2015) and how people with aphasia interact with others (Wilkinson, 2014).

From SIT research, it follows that social policy should encourage people who have acquired an impairment or who are non‐apparently disabled to join disability community groups, to experience disability culture to recenter joy in that identity and to collectively act for social change. DP research exposes ableism and puts an onus on non‐disabled people to reconsider how they understand and ‘talk up’ disability. This would reduce the ableism we (disabled people) experience every day and would address the disconnect between the joy cultivated by disabled communities and how society continues to position disability as a (medical) problem that resides in individual people, towards one that treats ableism in the same way as racism or sexism. Research‐wise, dialogue and integration between the different perspectives on disability in social psychology is now sorely needed. Cross‐perspective and emancipatory research that exposes the discrimination that disabled people experience supports disability rights movements (Pinto, 2019; see also Goodley & Moore, 2000; Shakespeare, 1996). As one of my recent papers (Jones, 2024) attests, raising awareness of these things is a start, but it is not enough. We need to help *everyone* become an agent of change, both to recognize the illegitimacy of ableism and to act to progress towards equality.

## CONCLUSIONS

This paper has briefly reviewed social psychology research, looking at ways in which ableist stereotypes may be addressed and that disabled joy may be encouraged. Using social identity research, it has been argued that cultivating a positive shared identity grounded in social and affirmative models of disability supports well‐being, disability pride and works towards collective action against ableism. Discursive social psychological research allows us to critically examine the voices and experiences of disabled people in emancipatory ways. At the same time, challenging stereotypes of disability that are in line with medical and deficit views of disability is likely to give both disabled and non‐disabled people agency to work towards social change and disabled people's rights.

## AUTHOR CONTRIBUTIONS

**Siân E. Jones:** Conceptualization; writing – original draft; writing – review and editing.

## CONFLICT OF INTEREST

The author has no conflict of interest to declare.

## Footnotes

^1^
Some disabilities are often labelled as ‘invisible’ or ‘hidden’, but disability activist and scholar Margaret Price (2011) instead distinguishes between ‘apparent’, ‘intermittently apparent’ and ‘non‐apparent’ disabilities. Describing disabilities as ‘invisible’ reflects a sight‐centric perspective on disability and access (which is arguably ableist). It also shifts responsibility away from the (non‐disabled) perceiver, placing the burden on disabled individuals to ‘disclose’ their identities (Price, 2011). I have a readily apparent physical impairment and am neurodivergent.
